# Establishment and Validation of Novel Clinical Prognosis Nomograms for Luminal A Breast Cancer Patients with Bone Metastasis

**DOI:** 10.1155/2020/1972064

**Published:** 2020-12-08

**Authors:** QiHao Tu, Chuan Hu, Hao Zhang, Chen Peng, Meng Kong, MengXiong Song, Chong Zhao, YuJue Wang, Jianyi Li, ChuanLi Zhou, Chao Wang, XueXiao Ma

**Affiliations:** ^1^Department of Orthopaedic Surgery, The Affiliated Hospital of Qingdao University, Qingdao 266071, China; ^2^Medical College of Qingdao University, Qingdao, 266000 Shandong, China

## Abstract

**Purpose:**

Overall survival (OS) and cancer-specific survival (CSS) of luminal A breast cancer (BC) patients with bone metastasis remain poor and vary dramatically from person to person. Our goal was to build two universally applicable nomograms to accurately predict OS and CSS for luminal A patients with bone metastasis.

**Methods:**

The data were collected from the Surveillance, Epidemiology, and End Results (SEER) database for luminal A BC patients with bone metastasis between 2010 and 2015. Univariate and multivariate Cox regression analyses were to assess and identify independent risk factors of OS and CSS. Integrating all significant predictors, nomograms and risk group stratification model was developed. The performance of the nomogram was validated with concordance index (C-index), calibration plots, and decision curve analyses (DCA) for discriminative ability, calibration, and clinical utility, respectively.

**Results:**

3171 luminal A BC patients with bone metastasis were included. Through univariate and multivariate Cox regression analyses, 12 variables were identified as both independent OS- and CSS-related factors, including age, race, primary site, histology grade, tumor size, surgery, brain metastasis, liver metastasis, lung metastasis, estrogen receptor status, progesterone receptor status, and insurance. Our nomograms for 1-, 3-, and 5-year survival were based on those significant prognostic factors to develop. The C-indexes of OS- and CSS-nomograms in the training cohort were 0.701 and 0.704, respectively. Similar results were obtained in the validation cohort. The calibration curves and DCA presented satisfactory calibration and clinical utility.

**Conclusion:**

Two nomograms have good discrimination, calibration, and clinical utility, can accurately and effectively predict the prognosis of patients, and may benefit for clinical decision-making. In high-risk patients, more aggressive therapy and closer surveillance should be considered.

## 1. Background

Breast cancer (BC) is the second most diagnosed cancer (11.6% of the cancer cases), second only to lung cancer, and accounts for a quarter of all female cancer cases [1]. Among females, BC is not only the most generally diagnosed cancer but also the main cause of cancer death [1]. The well-known classification criteria of breast cancer are depending on the status of molecular markers ER (estrogen receptor), PR (progesterone receptor), Ki-67, and Her2 (human epidermal growth factor receptor 2) [2]. BCs can be divided into molecular subtypes of Triple negative, luminal A, Luminal B, and HER2, with luminal A subtype being the most common one [3].

The main cause of death for BC patients is not the primary tumor but the occurrence of distant metastasis [4]. A cancer statistic among Hispanics/Latinos showed approximately over 30% of BC patients would have distant nonnodal metastases [5]. A population-based research including about 300,000 patients indicated that the bone metastasis (3.28%) takes the leading place in distant metastasis secondary to BC, which will develop in almost 3/4 of stage-IV BC patients [6], negatively affecting the patient's mobility, survival expectancy, and life quality. Poor prognosis is largely caused by skeletal-related events (SRE), mainly presenting as severe pain, pathologic fractures, spinal cord compression, and hypercalcemia [7, 8].

Indeed, tumor treatment has made great progress as medical technology further develops. However, accurate prediction and standard treatments for luminal A subtype BC patients with bone metastasis are lacking. Moreover, limitations of conventional predictors including RPA/GPA classification and TMN stage, old risk grouping, regression tree analyses, or probability tables have gradually been revealed. To supply the most appropriate and feasible clinical treatment, there is an urgent need for a convenient and effective tool to accurately predict prognosis. With advantages compared to old predictors, nomograms have been applied effectively for a long time in outcomes predicting based on data collected from clinics and laboratories [9, 10]. Furthermore, in several disciplines, studies, which compared different models have shown that nomograms based upon univariate and multivariate Cox regression are superior to other methodologies [11]. As a popular and effective prediction model, nomogram enables clinicians to evaluate the prognosis and choose an optimized treatment plan. The study is to develop new nomograms to predict the prognosis of luminal A subtype BC patients with bone metastasis.

## 2. Methods

### 2.1. Patient Selection and Grouping

The demographics, clinical and laboratory information of the luminal A patients with bone metastasis in the Surveillance, Epidemiology, and End Results (SEER) database from 2010 to June 2015 were collected. There is no need for informed consent in our study since the unidentified data was free from medical ethics review. The inclusion criteria were as follows: (1) patients diagnosed by immunohistochemistry; (2) patients with primary luminal A BC; (3) patients with bone metastasis. The exclusion criteria were as follows: (1) under 18 years old when diagnosed; (2) follow-up time < 2 months; (3) key information lacking. Finally, 3171 patients were selected and randomly allocated into two groups by R software with a ratio of 7 : 3. Descriptive statistics were used to summarize the status and the features of the training and validation datasets.

### 2.2. Variable Selection and Declaration

Following variables were selected in our study: follow time, tumor size, race, gender, age at diagnosis, primary site, grade, laterality, histologic type, T, N stage, treatment, metastasis, cause of death, status of life, ER, PR, treatment marital, and insurance. Some variables were analyzed and adjusted considering their type, performance, and occurrence rates in clinical manifestation, as well as the actual data volume. Tumor size was regrouped into three subcollections (below 36 mm, 36-85 mm, and above 85 mm). Patients were then divided into three subcollections based on their ages (<55, 55-78, and >78). Race was classified as white, black, or others. Histology was divided as infiltrating duct carcinoma (IDC), infiltrating duct and lobular carcinoma (IDC + ILC), or others. The primary site was stratified as lower-inner, upper-inner, or lower-outer quadrant of the breast, central portion of the breast, breast NOS, and others. T stage was regrouped into two subgroups (T1-2 and T3-4). N stage was regrouped into two subgroups (N0 and N1-3).

### 2.3. Nomogram Development and Statistical Analyses

Patients who had no ending events during the follow-up were also included in the analysis. OS or CSS was recognized as the endpoint of the study. OS was calculated from diagnosis to death led by any cause or the end of follow-up while CSS was from diagnosis to death caused by cancer [12]. Baseline characteristics comparison was performed by chi-square test, and risk factors for OS or CSS were evaluated by univariate Cox regression. Multivariate Cox regression was then conducted based on the results of univariate analysis. As a set of independent prognostic factors get screened, the nomograms for the OS and CSS of 1, 3, and 5 years were further constructed.

Aiming at the most simplified model with the strongest predicting capability, we conducted the establishment of the model under rigorous programmable decision so that its building procedure could get internally validated [13]. Meanwhile, we validated the models internally with the 1000 bootstrap resamples and conducted external validation on the validation cohort. The level of discrimination in this cohort was quantified and measured using the concordance index (C-index) and its 95% confidence interval (95% CI). The model's distinguishing ability improves when its C-index increases from 0.5 to 1.The maximum value of the C-index is 1.0, which indicates the model's perfect ability in correctly discriminating outcome. The consistency of the predicted results with the actual was further determined by the Calibration plot. Subsequently, the clinical utility of the nomogram was assessed by decision curve analysis (DCA) by quantifying quantified net benefits under various threshold probabilities [14], which was decided by the difference between the expected benefit and expected lose in association with every treatment strategy and proposed testing [15]. The patients of the training and validation datasets were categorized into groups of high-risk or low-risk in line with their nomogram-derived risk scores. The survival curve upon a log-rank test was used to evaluate the utility of nomogram in prognosis predicting. R software (http://www.r-poject.org, version 3.6.1) and the IBM SPSS 25.0 software were applied for all statistical analyses as above.

## 3. Results

### 3.1. Grouping and Baseline Characteristics

**Figure 1:**
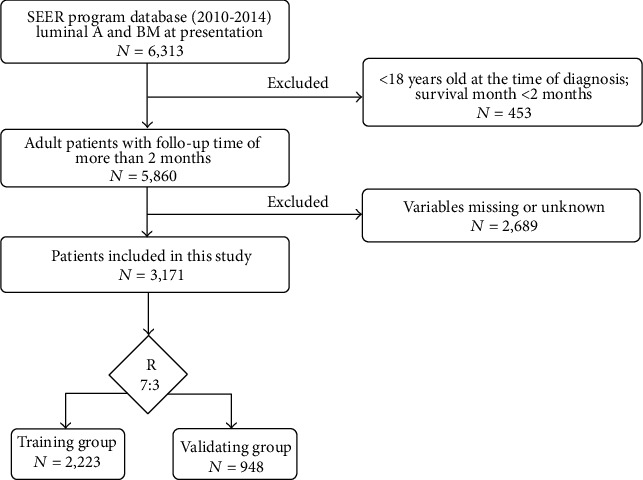
Flowchart of patient selection.

The flow chart of the process of patient inclusion, exclusion, and grouping is shown in Figure 1. According to the criteria in the method, 3,171 BC patients with bone metastasis were finally obtained. The R software was used to randomly divide all patients into training group (*N* = 2,223) and validation group (*N* = 948) at a ratio of 7 : 3. Mean age and follow time of all were 60 years old (range, 21–97) and 30.2 months. In terms of race, 78.4% (*n* = 2,485), 13.7% (*n* = 435), and 7.9% (*n* = 251) of patients were white, black, and other races, respectively. The most general histological type was infiltrating duct carcinoma (IDC) (*n* = 2,285, 72.1%). Moderate differentiation (Grade II) (*n* = 1,710, 53.9%) accounted for more than half proportion, with poor differentiation (Grade III-IV) (*n* = 1048, 33.0%) and good differentiation (Grade I) (*n* = 413, 13.0%) following. Regarding size, the majority of patients have a tumor size greater than 20 mm (*n* = 2,772, 71.6%). Among the socioeconomic factors, only a few patients have no insurance (*n* = 118, 3.7%). In the training group, almost half of all patients received chemotherapy (*n* = 1,058, 47.6%). Only 849 patients (38.2%) received surgery, 962 patients (43.3%) receiving radiotherapy. Detailed demographics and clinical information of the training and validation groups were summarized in Table 1.

### 3.2. Confirmation of Prognostic Factors and Development Nomograms

**Figure 2:**
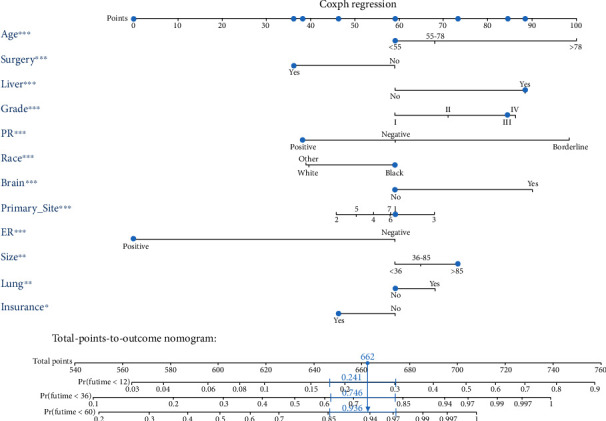
The novel nomogram to predict 1-, 3-, and 5-year overall survival of luminal A BC patients with bone metastasis.

**Figure 3:**
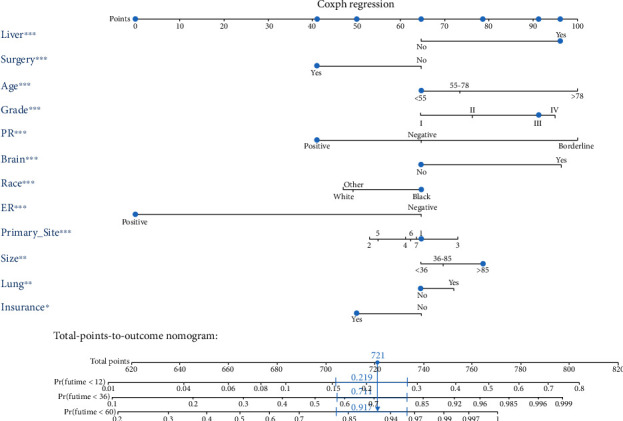
The novel nomogram to predict 1-, 3-, and 5-year cancer-specific survival of luminal A BC patients with bone metastasis.

We first conducted a univariate analysis to screen for relevant significant variables. The results of the univariate analysis on the training group can be viewed in Table 2. We obtained the significant *P* value, HR (hazards ratio), and 95% confidence intervals (CI) of the relative importance of each independent variable, including demographic, clinical, and socioeconomic factors. Subsequently, we conducted a multivariate Cox regression analysis of significant variables. Through univariate and multivariate Cox regression analyses, 12 independent variables, in significant association with OS and CSS, were identified including age, race, histology grade, tumor size, primary site, surgery, brain metastasis, liver metastasis, lung metastasis, ER status, PR status, and insurance. The results of the multivariate Cox regression analysis of OS and CSS on the training group are shown in Table 3. Ultimately, the significant variables mentioned above were included to build the nomogram. The nomograms of OS and CSS are shown in Figures 2 and 3. Nomogram is a quite user-friendly predictive tool which enables clinician or patient to determine the survival probability by calculating the scores of covariate and then draw a line vertically downward [16]. The scores assigned to each factor were listed in Table 4.

### 3.3. Nomogram Validation and Risk Stratification

**Figure 4:**
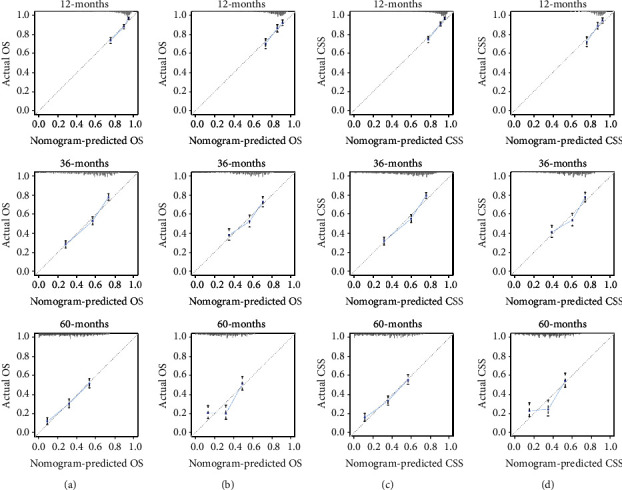
Calibration curves for predicting patients overall survival at 1-, 3-, and 5- years in the training cohort (a) and validation cohort (b). Calibration curves for predicting patient cancer-specific survival at 1-, 3-, and 5- years in the training cohort (c) and validation cohort (d).

**Figure 5:**
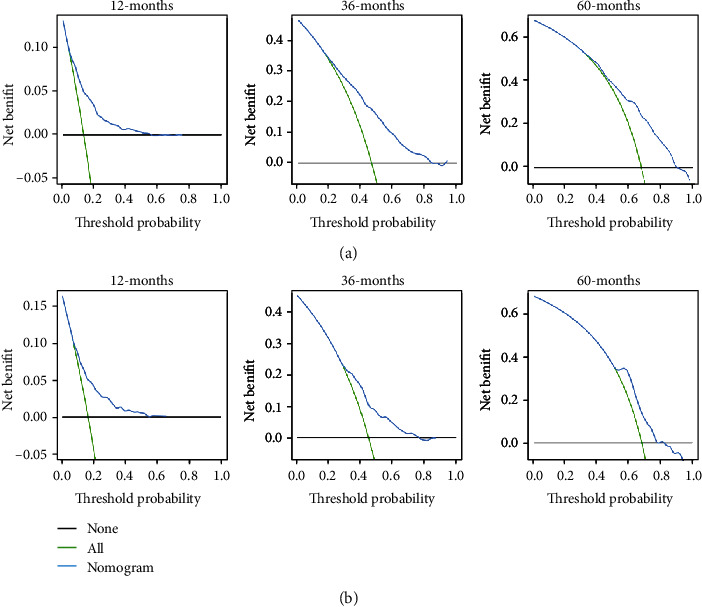
The decision curve analysis of the nomogram for predicting 1-, 3-, and 5-year overall survival in the training cohort (a) and in the validation cohort (b).

**Figure 6:**
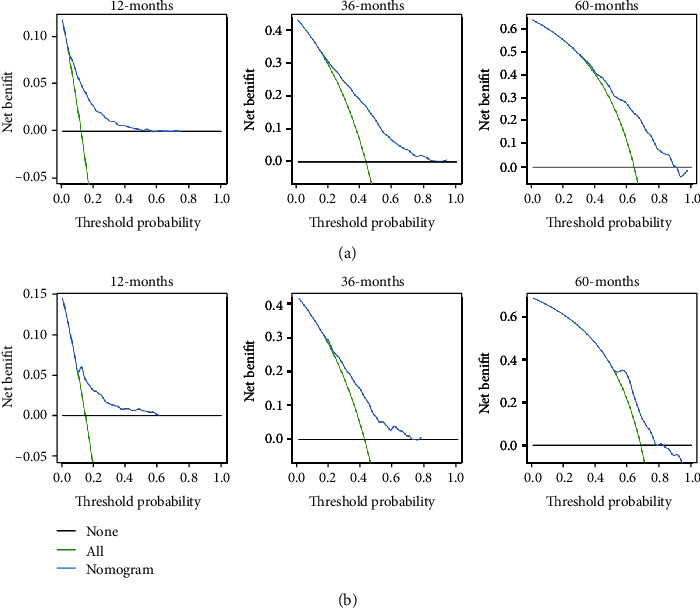
The decision curve analysis of the nomogram for predicting 1-, 3-, and 5-year cancer-specific survival in the training cohort (a) and in the validation cohort (b).

**Figure 7:**
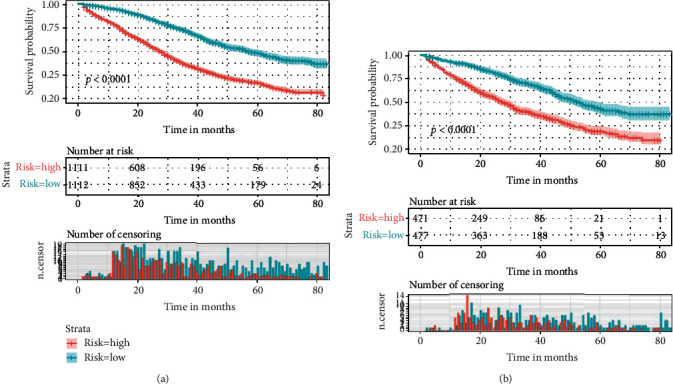
The Kaplan-Meier survival curve of risk group stratification for overall survival in the training cohort (a) and in the validation cohort (b).

**Figure 8:**
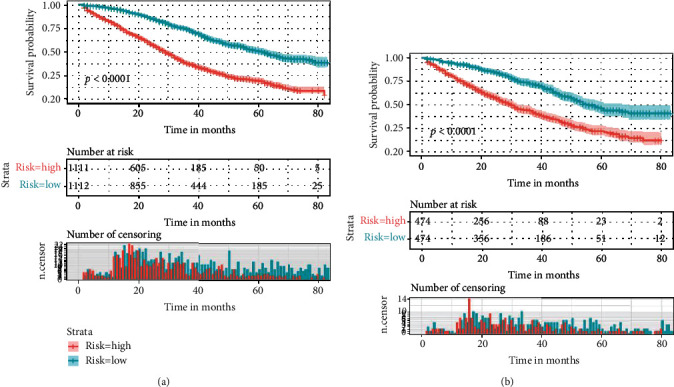
The Kaplan-Meier survival curve of risk group stratification for cancer-specific survival in training cohort (a) and in the validation cohort (b).

The performance of the nomograms was validated with C-index, calibration plots, and DCA for discriminative ability, accurate prediction, and clinical utility, respectively. The C-index of this model in the training group was 0.701 (95% CI: 0.688–0.720) for the OS model and 0.704 (95% CI, 0.688–0.720) for the CSS model. In the validation group, the C-index of OS was 0.665(95% CI: 0.63–0.692), while that of CCS was 0.678 (95% CI, 0.651–0.705), underlying the good discriminating ability of the nomograms in the training and verification group. The prediction curves of OS and CSS in the training and validation groups at 1, 3, and 5 years were close to the standard curve (*Y* = *X*), indicating that the prediction results of nomograms have a significant correlation with the actual observation (Figure 4). DCA of 1-, 3-, and 5-year OS and CSS showed that the nomograms had a higher net benefit in the training cohort and validation cohort, respectively (Figures 5 and 6). According to our OS nomogram and CSS nomogram, risk scores were calculated for each luminal A patient with bone metastasis. In addition, it has been tested by the Kaplan-Meier survival curve that patients of the low-risk group present better prognosis than those in the high-risk group (Figures 7 and 8).

## 4. Discussion

For patients of BC with bone metastasis, the long-term survival and life quality in the later period are still not optimistic, and yet convenient and accurate prognostic predicting tool lacks. Recent studies point that the prediction ability of nomograms may be superior to that of traditional, categorical predictive models for various outcomes associated with cancer [17–19]. To take a step further, we performed the first large-cohort and comprehensive retrospective study based on wide multicenter, where the OS and the CSS of luminal A patients with bone metastasis (*n* = 3,171) selected from the SEER database were retrospectively analyzed. Through univariate and multivariate Cox regression analyses, 12 independent variables associated with the OS and CSS were finally identified. Two nomograms established based on these significant prognosis predicting indicators showed high levels of discrimination and calibration in clinical utility.

Although BC bone metastasis is still incurable, our survival curves showed that the survival probability for patients of the low-risk group is significantly higher than those of the high-risk group. Therefore, it appears to be crucial to identify the risk factors for facilitating the prognosis prediction. Consistent with previous studies, our study suggests that age is a strong independent prognostic factor and young age is an advantageous factor for good prognosis [20, 21]. Besides, a report focusing on the OS time trends indicates that every incremental year of age is in independent and significant association with a higher risk of death [22]. On the contrary, old age may be a disadvantageous factor with poor status and age-related comorbidities. In addition, some targeted therapy or other intensive systemic treatment may be contraindicated to the old patients who are vulnerable to more frequent causes of death. In line with our conclusion, it has been previously reported that race is a significant survival predictor [23]. Parada et al. [24] pointed out that racial differences in gene expression might lead to the survival disparity of BC patients. Our study shows that insurance status is also a significant variable. In many states of the USA, health insurance not only compensated patients for surgery but also reduced the cost of systemic adjuvant treatments. And insurance status has shown its impact on stages of diagnosis in a previous study [25]. Moreover, Pan et al. [23] developed that in addition to the impact on diagnosis, uninsured status was also demonstrated to be an unfavorable factor of poor OS and CSS.

In our conclusion, tumor size, tumor primary site, and histology grade were recognized as risk factors of great importance in affecting the prognosis of BC patients, which were in accordance with previous studies [26–29]. With our regression analyses, brain, liver, and lung metastasis were also independent predictors of prognosis, among which brain metastasis was most likely to result in poor prognosis, followed by liver and lung metastasis. When considering all BC patients as an entire population, a cohort study has found that different distant metastatic sites presented similar trends in affecting survival [30]. Moreover, the effective implications of ER and PR status have been demonstrated by some large-scale studies in predicting patients' prognosis and responding to BC endocrine therapy [31, 32]. Seho et al. [33] also concluded that lack of expression of either ER or PR was in association with worse prognosis, especially among patients with node-positive luminal A subtype.

For cancer patients who have been metastasized, to perform surgery is still controversial. Similar to previous reports, our research showed that nonsurgical luminal A patients with bone metastasis had an unfavorable prognosis. Generally, surgical treatment for primary lesion is recognized as a palliative therapy for BC patients with metastasis. Gnerlich et al. [34] showed an association between receiving surgery and improved survival for BC patients with metastasis. Xiong et al. [35] pointed that the prognosis of certain stage IV BC patients, especially those with bone- or soft tissue-only metastasis, could be improved by surgical removal of primary lesions. Moreover, for BC patients with bone metastasis, surgery can not only prolong the survival time but also improve life quality to some extent. And it is generally believed that chemotherapy can exert a similar effect by reducing cancer-related complications through killing or inhibiting cancer cells, thereby relapse delayed and survival time prolonged. However, chemotherapy failed to be identified as a significant predictor for either OS or CSS in our multivariate analysis. In fact, our conclusion is not an exception with support of other studies, where no benefit of adjuvant chemotherapy was detected in luminal A BC patients [36, 37]. The Panel of the St. Gallen International Expert Consensus insisted that was less useful in Luminal A subtype patients for their less responsiveness to chemotherapy [38]. In addition, consistent with our results, a retrospective cohort study suggested that there was no significant effect of radiotherapy in improving survival of BC with metastasis [39].

To our knowledge, this study is the first to construct comprehensive nomograms to predict the prognosis of Luminal A BC patients with bone metastasis. Our nomograms were based on twelve independent and significant prognosis factors selected from univariate and multivariate Cox regression analyses with satisfied level of discrimination, calibration, and clinical utility, which can help predict the survival probability and expected benefits of different treatments, so that the most suitable one can be selected and the prognosis can get improved. In addition, there are many kinds of predictors included in our nomograms, implying that the luminal A with bone metastasis is a complex disease with considerable individual differences. In recent years, against the increasing emphasis on personalization of cancer treatment strategies, our nomograms can make accurate individualized predictions for each luminal A subtype patient with bone metastasis. By adding the scores of each variable, the physician can clearly assess the prognosis of the patient. Combined with the evaluation results, for high-risk patients, more appropriate treatments and more optimized care can be provided. In contrast, for low-risk patients, some treatment and examinations can be appropriately adjusted or reduced, thereby lessening the patient's physical and economic burden.

However, there are several limitations in the present research. First, our nomograms were based on a retrospective cohort obtained from SEER-base, which inevitably creates bias. Second, the data may lack some potentially important variables and key indicators, such as related biomarkers, hormone therapy, targeted therapy, recurrence, and other advanced technologies. Third, some data are missing or not in detail, especially specific locations of bone metastasis and types of surgery. These deficiencies remain to be further improved in future studies.

## 5. Conclusions

Our study identified twelve independent prognostic factors for OS and CSS of luminal A BC patients with bone metastasis. The nomograms we developed can accurately and effectively predict the survival information of patients and may facilitate clinical decision-making.

## Figures and Tables

**Table 1:** Demographic, clinical, and laboratory features of patients diagnosed as luminal A with bone metastasis.

Variable	Training set (*n* = 2,223)	Validating set (*n* = 948)	*P* value
*Follow time (mo)*			
Mean	30.4	29.8	
Range	2-83	2-83	
*Number of events*			
Live	1059 (47.6%)	462 (48.7%)	
Dead	1164 (52.4%)	486 (51.3%)	
*Age (y)*			0.387
<55	776 (34.9%)	307 (32.4%)	
55-78	1220 (54.9%)	539 (56.9%)	
>78	227 (10.2%)	102 (10.8%)	
*Race*			0.748
White	1743 (78.4%)	742 (78.3%)	
Black	300 (13.5%)	135 (14.2%)	
Other	180 (8.1%)	71 (7.5%)	
*Grade*			0.340
I	292 (13.1%)	121 (12.8%)	
II	1190 (53.5%)	520 (54.9%)	
III	734 (33.0%)	307 (32.4%)	
IV	7 (0.3%)	0	
*Laterality*			0.260
Left	1138 (51.2%)	474 (50.0%)	
Right	1085 (48.8%)	473 (49.9%)	
Bilateral	0	1 (0.01%)	
*Histological type*			0.731
IDC	1607 (72.3%)	678 (71.5%)	
IDC + ILC	136 (6.1%)	65 (6.9%)	
Other	480 (21.6%)	205 (21.6%)	
*T stage*			0.806
T1-2	1183 (53.2%)	509 (53.7%)	
T3-4	1040 (46.8%)	439 (46.3%)	
*N stage*			0.867
N0	522 (23.5%)	220 (23.2%)	
N1-3	1701 (76.5%)	728 (76.8%)	
*Size (mm)*			0.083
<36	973 (43.8%)	454 (47.9%)	
36-85	1019 (45.8%)	396 (41.8%)	
>85	231 (10.4%)	98 (10.3%)	
*Primary site*			0.134
Breast, NOS	487 (21.9%)	252 (26.6%)	
Central portion of breast	180 (8.1%)	66 (7.0%)	
Lower-inner quadrant of breast	94 (4.2%)	41 (4.3%)	
Lower-outer quadrant of breast	140 (6.3%)	55 (5.8%)	
Upper-inner quadrant of breast	179 (8.1%)	64 (6.8%)	
Upper-outer quadrant of breast	619 (27.8%)	248 (26.2%)	
Other	524 (23.6%)	222 (23.4%)	
*Surgery*			0.734
Yes	849 (38.2%)	356 (37.6%)	
No	1374 (61.8%)	592 (62.4%)	
*Radiation*			<0.0001
Yes	962 (43.3%)	516 (54.4%)	
No	1261 (56.7%)	432 (45.6%)	
*Chemotherapy*			0.322
Yes	1058 (47.6%)	433 (45.7%)	
No	1165 (52.4%)	515 (54.3%)	
*Brain*			0.009
Yes	103 (4.6%)	25 (2.6%)	
No	2120 (95.4%)	923 (97.4%)	
*Liver*			0.026
Yes	347 (15.6%)	119 (12.6%)	
No	1876 (84.4%)	829 (87.4%)	
*Lung*			0.947
Yes	469 (21.1%)	199 (21.0%)	
No	1754 (78.9%)	749 (79.0%)	
*ER*			0.097
Positive	2208 (99.3%)	936 (98.7%)	
Negative	15 (0.7%)	12 (1.3%)	
*PR*			0.973
Positive	1903 (85.6%)	813 (85.8%)	
Negative	317 (14.3%)	134 (14.1%)	
Borderline	3 (0.1%)	1 (0.1%)	
*Insurance*			0.007
Yes	2127 (95.7%)	926 (97.7%)	
No	96 (4.3%)	22 (2.3%)	
*Marital status*			0.004
Yes	1127 (50.7%)	428 (45.1%)	
No	1096 (49.3%)	520 (54.9%)	

**Table 2:** Univariate Cox regression analysis of overall survival and cancer-specific survival in the training group.

Variable	OS			CSS		
	HR	95% CI	P	HR	95% CI	P
*Age (y)*						
<55	Reference			Reference		
55-78	1.230	1.082-1.399	0.002	1.215	1.045-1.413	0.011
>78	2.187	1.812-2.639	0.000	1.922	1.542-2.397	0.000
*Size (mm)*						
<36	Reference			Reference		
36-85	1.154	1.020-1.305	0.023	1.143	1.004-1.302	0.043
>85	1.601	1.327-1.933	0.000	1.644	1.351-1.999	0.000
*Race*						
Black	Reference			Reference		
Other	0.614	0.478-0.790	0.000	0.667	0.514-0.866	0.002
White	0.575	0.491-0.672	0.000	0.590	0.499-0.697	0.000
*Primary site*						
Breast, NOS	Reference			Reference		
Central portion	0.670	0.522-0.860	0.002	0.684	0.527-0.888	0.004
Lower-outer quadrant	0.803	0.677-0.952	0.011	0.788	0.659-0.943	0.009
*Grade*						
I	Reference			Reference		
II	1.319	1.082-1.607	0.006	1.301	1.056-1.603	0.014
III	1.885	1.539-2.309	0.000	1.937	1.564-2.398	0.000
IV	2.478	1.091-5.628	0.030	2.746	1.206-6.252	0.016
*T stage*						
T1,T2	Reference			Reference		
T3,T4	1.299	1.157-1.457	0.000	1.288	1.141-1.454	0.000
*Surgery*						
No	Reference			Reference		
Yes	0.539	0.476-0.609	0.000	0.533	0.468-0.608	0.000
*Chemotherapy*						
No	Reference					
Yes	0.859	0.765-0.964	0.010			
*Brain metastasis*						
No	Reference			Reference		
Yes	2.506	1.996-3.147	0.000	2.607	2.060-3.299	0.000
*Liver metastasis*						
No	Reference			Reference		
Yes	2.174	1.884-2.509	0.000	2.330	2.010-2.701	0.000
*Lung metastasis*						
No	Reference			Reference		
Yes	1.666	1.463-1.898	0.000	1.642	1.431-1.884	0.000
*ER*						
Negative	Reference			Reference		
Positive	0.244	0.141-0.423	0.000	0.219	0.127-0.380	0.000
*PR*						
Negative	Reference			Reference		
Positive	0.551	0.473-0.641	0.000	0.517	0.442-0.604	0.000
*Insurance*						
No	Reference			Reference		
Yes	0.721	0.558-0.931	0.012	0.681	0.524-0.886	0.004
*Marital status*						
No	Reference			Reference		
Yes	0.770	0.686-0.864	0.000	0.807	0.715-0.911	0.001

**Table 3:** Multivariate Cox regression analysis of overall survival and cancer-specific survival in the training group.

Variable	OS			CSS		
	HR	95% CI	P	HR	95% CI	P
*Age (y)*						
<55	1			1		
55-78	1.218	1.068-1.390	0.003	1.204	1.031-1.405	0.019
>78	2.476	2.039-3.006	0.000	2.137	1.697-2.692	0.000
*Size (mm)*						
<36	1			1		
36-85	1.131	0.998-1.282	0.053	1.078	0.925-1.257	0.337
>85	1.363	1.118-1.661	0.002	1.292	1.014-1.645	0.038
*Race*						
Black	1			1		
Other	0.650	0.503-0.839	0.001	0.724	0.553-0.946	0.018
White	0.641	0.546-0.752	0.000	0.686	0.578-0.815	0.000
*Primary site*						
Breast, NOS	1			1		
Central portion	0.744	0.578-0.958	0.022	0.767	0.589-0.998	0.048
Lower-inner quadrant	1.021	0.825-1.264	0.846	1.044	0.834-1.308	0.706
Lower-outer quadrant	0.823	0.693-0.978	0.027	0.811	0.676-0.974	0.025
Other	0.975	0.833-1.142	0.752	0.980	0.829-1.158	0.811
*Grade*						
I	1			1		
II	1.297	1.063-1.583	0.010	1.293	1.048-1.597	0.017
III	1.755	1.425-2.160	0.000	1.806	1.450-2.249	0.000
IV	1.817	0.789-4.182	0.160	1.949	0.843-4.506	0.119
*T stage*						
T1,T2				1		
T3,T4				1.061	0.906-1.241	0.463
*Surgery*						
No	1			1		
Yes	0.601	0.528-0.683	0.000	0.593	0.518-0.680	0.000
*Brain metastasis*						
No	1			1		
Yes	1.977	1.560-2.506	0.000	2.003	1.566-2.562	0.000
*Liver metastasis*						
No	1			1		
Yes	1.910	1.643-2.221	0.000	2.016	1.726-2.355	0.000
*Lung metastasis*						
No	1			1		
Yes	1.219	1.063-1.398	0.005	1.185	1.026-1.370	0.021
*ER*						
Negative	1			1		
Positive	0.272	0.156-0.477	0.000	0.235	0.134-0.413	0.000
*PR*						
Negative	1			1		
Positive	0.634	0.543-0.740	0.000	0.587	0.501-0.689	0.000
Borderline	2.336	0.739-7.388	0.149	2.167	0.683-6.877	0.189
*Insurance*						
No	1			1		
Yes	0.755	0.581-0.980	0.035	0.727	0.556-0.950	0.020
*Marital*						
No				1		
Yes				0.916	0.807-1.040	0.176

**Table 4:** Value assignment of the independent prognostic factors contained in the OS-and CSS-nomograms.

Prognostic factors	OS	CSS
*Age (y)*		
<55	59	65
55-78	68	73
>78	100	100
*Size (mm)*		
<36	59	65
36-85	65	70
>85	73	79
*Race*		
*Black*	59	65
Other	40	49
White	39	47
*Primary site*		
Breast, NOS	59	65
Central portion of breast	46	53
Lower-inner quadrant of breast	68	73
Lower-outer quadrant of breast	54	61
Upper-inner quadrant of breast	58	62
Upper-outer quadrant of breast	58	64
Other	50	55
*Grade*		
I	59	65
II	71	76
III	85	91
IV	86	95
*Surgery*		
No	59	65
Yes	36	41
*Brain metastasis*		
No	59	65
Yes	90	96
*Liver metastasis*		
No	59	65
Yes	88	96
*Lung metastasis*		
No	59	65
Yes	68	72
*ER*		
Negative	59	65
Positive	0	0
*PR*		
Negative	59	65
Positive	38	41
Borderline	98	100
*Insurance*		
No	59	65
Yes	46	50

## Data Availability

The data analyzed during the study are available from the SEER data set repository and/or authors.

## Abbreviations

BC: Breast cancer
OS: Overall survival
CSS: Cancer-specific survival
SEER: Surveillance, epidemiology, and end results
ER: Estrogen receptor
PR: Progesterone receptor
Her2: Human epidermal growth factor receptor 2
C-index: Concordance index
DCA: Decision curve analysis.
